# 3-Hydr­oxy-4-methoxy­benzaldehyde thio­semicarbazone hemihydrate

**DOI:** 10.1107/S1600536808035617

**Published:** 2008-11-08

**Authors:** Hoong-Kun Fun, Reza Kia, E. Deepak D’Silva, P. S. Patil, S. M. Dharmaprakash

**Affiliations:** a X-ray Crystallography Unit, School of Physics, Universiti Sains Malaysia, 11800 USM, Penang, Malaysia; b Department of Studies in Physics, Mangalore University, Mangalagangotri, Mangalore 574 199, India; c Department of Physics, KLE Society’s Institute of Technology, Gokul Road, Hubli 590 030, India

## Abstract

The asymmetric unit of the title compound, C_9_H_11_N_3_O_2_S·0.5H_2_O, comprises two crystallograpically independent thio­semicarbazone mol­ecules (*A* and *B*) and a water mol­ecule of crystallization. In each of the thio­semicarbazone mol­ecules, intra­molecular O—H⋯O and N—H⋯N hydrogen bonds form five-membered rings, producing *S*(5) ring motifs. Inter­molecular O—H⋯S and N—H⋯O inter­actions between mol­ecule *B* and the water mol­ecule form a six-membered ring, producing an *R*_2_^2^(6) ring motif. Inter­molecular N—H⋯S hydrogen bonds form dimers involving pairs of both *A* and *B* mol­ecules, which form *R*_2_^2^(8) ring motifs. The angles between the aromatic ring and thio­urea unit in the two mol­ecules are 0.80 (6) and 3.28 (5)°, which proves that each mol­ecule is fairly planar. The crystal structure is stabilized by inter­molecular O—H⋯S (×2), O—H⋯O, N—H⋯S (×2) and N—H⋯O (×2) hydrogen bonds and C—H⋯O (×2) contacts to form a three-dimensional network.

## Related literature

For details of hydrogen-bond motifs, see: Bernstein *et al.* (1995 ▶). For background to thio­semicarbazones, see: Al-Awadi *et al.* (2008 ▶); Kizilcikli *et al.* (2004 ▶); Mishra *et al.* (2006 ▶). For a related structure, see: Ferrari *et al.* (2001 ▶).
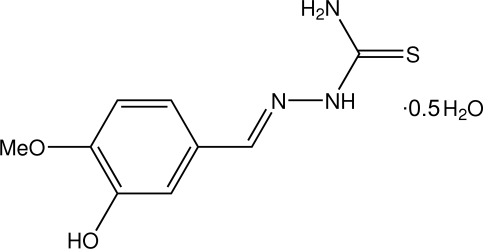


## Experimental

### 

#### Crystal data

C_9_H_11_N_3_O_2_S·0.5H_2_O
*M* *_r_* = 234.28
Triclinic, 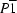

*a* = 10.5288 (2) Å
*b* = 10.7045 (2) Å
*c* = 11.8154 (2) Å
α = 68.438 (1)°
β = 68.917 (1)°
γ = 68.114 (1)°
*V* = 1110.28 (4) Å^3^
*Z* = 4
Mo *K*α radiation
μ = 0.28 mm^−1^
*T* = 100.0 (1) K
0.45 × 0.32 × 0.10 mm

#### Data collection

Bruker SMART APEXII CCD area-detector diffractometer
Absorption correction: multi-scan (**SADABS**; Bruker, 2005 ▶) *T*_min_ = 0.884, *T*_max_ = 0.973
45467 measured reflections
10830 independent reflections
8078 reflections with *I* > 2σ(*I*)
*R*_int_ = 0.040

#### Refinement

*R*[*F* ^2^ > 2σ(*F* ^2^)] = 0.042
*wR*(*F* ^2^) = 0.116
*S* = 1.10
10830 reflections
314 parameters
H atoms treated by a mixture of independent and constrained refinement
Δρ_max_ = 0.90 e Å^−3^
Δρ_min_ = −0.63 e Å^−3^

### 

Data collection: *APEX2* (Bruker, 2005 ▶); cell refinement: *APEX2*; data reduction: *SAINT* (Bruker, 2005 ▶); program(s) used to solve structure: *SHELXTL* (Sheldrick, 2008 ▶); program(s) used to refine structure: *SHELXTL*; molecular graphics: *SHELXTL*; software used to prepare material for publication: *SHELXTL* and *PLATON* (Spek, 2003 ▶).

## Supplementary Material

Crystal structure: contains datablocks global, I. DOI: 10.1107/S1600536808035617/tk2321sup1.cif

Structure factors: contains datablocks I. DOI: 10.1107/S1600536808035617/tk2321Isup2.hkl

Additional supplementary materials:  crystallographic information; 3D view; checkCIF report

## Figures and Tables

**Table 1:** Hydrogen-bond geometry (Å, °)

*D*—H⋯*A*	*D*—H	H⋯*A*	*D*⋯*A*	*D*—H⋯*A*
O1*W*—H2*W*1⋯S1*B*	0.86	2.28	3.1257 (9)	169
O1*W*—H1*W*1⋯O1*A*^i^	0.85	1.95	2.7955 (12)	173
N2*A*—H2N*A*⋯S1*A*^ii^	0.929 (18)	2.450 (18)	3.3732 (9)	172.6 (18)
N2*B*—H2N*B*⋯S1*B*^iii^	0.842 (19)	2.571 (18)	3.4055 (10)	171.2 (18)
N3*A*—H3N*A*⋯N1*A*	0.847 (19)	2.258 (15)	2.6129 (14)	105.4 (12)
N3*A*—H3N*B*⋯O1*W*^iv^	0.864 (16)	2.000 (15)	2.8408 (12)	164.0 (17)
N3*B*—H3N*C*⋯O1*W*	0.833 (19)	2.399 (19)	3.1492 (14)	150.2 (17)
N3*B*—H3N*D*⋯N1*B*	0.875 (19)	2.288 (17)	2.6554 (14)	105.2 (13)
O1*A*—H1O*A*⋯O2*A*	0.81 (2)	2.185 (18)	2.6292 (12)	114.5 (15)
O1*B*—H1O*B*⋯S1*A*^v^	0.82 (2)	2.685 (19)	3.2346 (10)	125.9 (16)
O1*B*—H1O*B*⋯O2*B*	0.82 (2)	2.251 (19)	2.6949 (13)	114.4 (16)
C1*B*—H1*BA*⋯O1*W*^vi^	0.93	2.40	3.3140 (14)	169
C9*B*—H9*BA*⋯O2*A*^vii^	0.96	2.51	3.2286 (15)	131

Symmetry codes: (i) 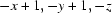; (ii) 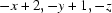; (iii) 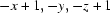; (iv) 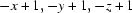; (v) 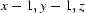; (vi) 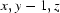; (vii) 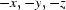.

**Fig. 1.**
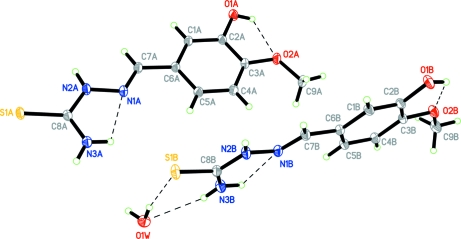
The molecular structure of (I) showing 50% probability displacement ellipsoids and the atomic numbering. Dashed lines show intramolecular hydrogen bonds.

**Fig. 2.**
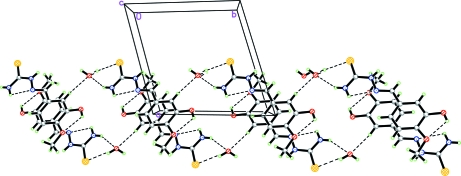
Partial crystal packing in (I), viewed down the c-axis, showing 1-D chains mediated by the water molecule along the b-axis. Intra- and inter-molecular interactions are drawn as dashed lines.

## supplementary crystallographic information

### Comment

Intriguing chelating patterns, biomedical properties, structural diversity and ion-sensing abilities (Al-Awadi *et al.*, 2008; Kizilcikli *et al.*, 2004; Mishra *et al.*, 2006) have made thiosemicarbazones a class of compounds of immense importance. We report herein the crystal structure of the title compound, (I).

The bond lengths and angles in (I), Fig. 1, agree with those in a related structure (Ferrari *et al.* 2001). Intramolecular O—H···O and N—H···N hydrogen bonds, in each molecule of A and B, form five-membered rings, producing *S*(5) ring motifs (Bernstein *et al.* 1995). The angle between the aromatic ring and the thiourea unit in each of molecule A and B is 0.80 (6) and 3.28 (5)°, respectively, which indicates each molecule is almost planar. Intermolecular O—H···S and N—H···O interactions between molecule B and the water molecule form a six-membered ring, producing a *R*_2_^2^(6) ring motif. Intermolecular N—H···S interactions for pairs of molecule A and similarly for pairs of molecules B lead to the formation of dimers with *R*_2_^2^(8) ring motifs (Bernstein *et al.* 1995). The crystal structure is stabilized by intermolecular O—H···S, O—H···O, N—H···S (*x* 2) and N—H···O (*x* 2) hydrogen bonds and C—H···O (*x* 2) contacts, see Table 1. In the 3-D crystal structure the water molecules link neghbouring molecules to form 1-D chains along the *b*-axis of the unit cell (Fig. 2).

### Experimental

3-Hydroxy-4-methoxy benzaldehyde (0.075 mol) and thiosemicarbazone (0.05 mol) were dissolved in a sufficient volume of methanol and the mixture was refluxed for 4 h until the whole volume of the mixture attains a pale-yellow colour. The mixture was then allowed to cool, poured into a beaker and kept aside for evaporation. The resulting crude sample was recrystallized twice from methanol. Pure light-yellow crystals of (I) were then obtained.

### Refinement

The H atoms of the water molecule were located from the difference Fourier map and constrained to refine on the parent atom with O—H = 0.85 - 0.86 Å, and with *U*(H) set to 1.5 times *U*_eq_(O). The H atoms bound to the remaining O and N atoms were located from a difference Fourier map and refined freely, see Table 1 for distances. The C-bound H atoms were positioned geometrically and refined in the riding model approximation with C—H = 0.93 - 0.96 Å, and with *U*(H) set to 1.2 - 1.5 times *U*_eq_(C). The rotating group model was applied to the methyl groups.

### Crystal data

**Table:**

C_9_H_11_N_3_O_2_S·0.5H_2_O	*Z* = 4
*M**_r_* = 234.28	*F*_000_ = 492
Triclinic, *P*1	*D*_x_ = 1.402 Mg m^−^^3^
Hall symbol: -P 1	Mo *K*α radiation λ = 0.71073 Å
*a* = 10.5288 (2) Å	Cell parameters from 9992 reflections
*b* = 10.7045 (2) Å	θ = 2.5–36.3º
*c* = 11.8154 (2) Å	µ = 0.28 mm^−^^1^
α = 68.438 (1)º	*T* = 100.0 (1) K
β = 68.917 (1)º	Plate, light yellow
γ = 68.114 (1)º	0.45 × 0.32 × 0.10 mm
*V* = 1110.28 (4) Å^3^	

### Data collection

**Table:**

Bruker SMART APEXII CCD area-detector diffractometer	10830 independent reflections
Radiation source: fine-focus sealed tube	8078 reflections with *I* > 2σ(*I*)
Monochromator: graphite	*R*_int_ = 0.040
*T* = 100.0(1) K	θ_max_ = 36.6º
φ and ω scans	θ_min_ = 2.1º
Absorption correction: multi-scan(*SADABS*; Bruker, 2005)	*h* = −17→17
*T*_min_ = 0.884, *T*_max_ = 0.973	*k* = −17→17
45467 measured reflections	*l* = −19→19

### Refinement

**Table:**

Refinement on *F*^2^	Secondary atom site location: difference Fourier map
Least-squares matrix: full	Hydrogen site location: inferred from neighbouring sites
*R*[*F*^2^ > 2σ(*F*^2^)] = 0.042	H atoms treated by a mixture of independent and constrained refinement
*wR*(*F*^2^) = 0.116	*w* = 1/[σ^2^(*F*_o_^2^) + (0.0547*P*)^2^ + 0.1125*P*] where *P* = (*F*_o_^2^ + 2*F*_c_^2^)/3
*S* = 1.10	(Δ/σ)_max_ = 0.001
10830 reflections	Δρ_max_ = 0.90 e Å^−^^3^
314 parameters	Δρ_min_ = −0.63 e Å^−^^3^
Primary atom site location: structure-invariant direct methods	Extinction correction: none

### Special details

**Table:**

Experimental. The low-temperature data was collected with the Oxford Cyrosystem Cobra low-temperature attachment.
Geometry. All e.s.d.'s (except the e.s.d. in the dihedral angle between two l.s. planes) are estimated using the full covariance matrix. The cell e.s.d.'s are taken into account individually in the estimation of e.s.d.'s in distances, angles and torsion angles; correlations between e.s.d.'s in cell parameters are only used when they are defined by crystal symmetry. An approximate (isotropic) treatment of cell e.s.d.'s is used for estimating e.s.d.'s involving l.s. planes.
Refinement. Refinement of *F*^2^ against ALL reflections. The weighted *R*-factor *wR* and goodness of fit *S* are based on *F*^2^, conventional *R*-factors *R* are based on *F*, with *F* set to zero for negative *F*^2^. The threshold expression of *F*^2^ > σ(*F*^2^) is used only for calculating *R*-factors(gt) *etc*. and is not relevant to the choice of reflections for refinement. *R*-factors based on *F*^2^ are statistically about twice as large as those based on *F*, and *R*- factors based on ALL data will be even larger.

### Fractional atomic coordinates and isotropic or equivalent isotropic displacement parameters (Å^2^)

**Table:**

	*x*	*y*	*z*	*U*_iso_*/*U*_eq_	
S1A	0.96703 (3)	0.48913 (3)	0.19973 (2)	0.01747 (6)	
O1A	0.51514 (8)	0.27402 (9)	−0.26347 (7)	0.02001 (15)	
O2A	0.30169 (8)	0.21411 (9)	−0.07261 (7)	0.01997 (15)	
N1A	0.71556 (9)	0.38149 (9)	0.09845 (8)	0.01559 (15)	
N2A	0.82582 (9)	0.42443 (9)	0.09440 (8)	0.01634 (15)	
N3A	0.72833 (10)	0.41202 (10)	0.30323 (9)	0.01863 (16)	
C1A	0.60929 (10)	0.32375 (11)	−0.13430 (9)	0.01602 (17)	
H1AA	0.6832	0.3453	−0.2035	0.019*	
C2A	0.50692 (10)	0.28289 (11)	−0.14735 (9)	0.01535 (16)	
C3A	0.39489 (10)	0.25053 (10)	−0.04411 (9)	0.01556 (16)	
C4A	0.38655 (10)	0.25938 (11)	0.07304 (9)	0.01677 (17)	
H4AA	0.3126	0.2376	0.1421	0.020*	
C5A	0.48947 (10)	0.30101 (11)	0.08621 (9)	0.01594 (17)	
H5AA	0.4835	0.3078	0.1643	0.019*	
C6A	0.60192 (10)	0.33279 (10)	−0.01652 (9)	0.01479 (16)	
C7A	0.71238 (10)	0.37674 (11)	−0.00763 (9)	0.01642 (17)	
H7AA	0.7819	0.4018	−0.0799	0.020*	
C8A	0.83058 (10)	0.43992 (10)	0.20092 (9)	0.01451 (16)	
C9A	0.18681 (12)	0.17340 (13)	0.02852 (11)	0.0237 (2)	
H9AA	0.1261	0.1552	−0.0041	0.036*	
H9AB	0.1337	0.2473	0.0688	0.036*	
H9AC	0.2236	0.0904	0.0886	0.036*	
S1B	0.47040 (3)	0.21971 (3)	0.47447 (3)	0.02029 (6)	
O1B	0.02689 (9)	−0.32448 (8)	0.32698 (8)	0.02095 (15)	
O2B	−0.18810 (8)	−0.11332 (8)	0.24783 (7)	0.01905 (14)	
N1B	0.20913 (9)	0.10835 (9)	0.38890 (8)	0.01597 (15)	
N2B	0.31944 (9)	0.10741 (9)	0.42677 (8)	0.01711 (15)	
N3B	0.23043 (10)	0.34380 (10)	0.39796 (9)	0.02002 (17)	
C1B	0.10696 (10)	−0.16764 (10)	0.36407 (9)	0.01606 (17)	
H1BA	0.1780	−0.2427	0.3942	0.019*	
C2B	0.01113 (10)	−0.19098 (10)	0.32466 (9)	0.01548 (16)	
C3B	−0.09831 (10)	−0.07809 (10)	0.28235 (9)	0.01488 (16)	
C4B	−0.10910 (10)	0.05636 (10)	0.27978 (9)	0.01613 (17)	
H4BA	−0.1822	0.1310	0.2526	0.019*	
C5B	−0.01134 (10)	0.07990 (10)	0.31756 (9)	0.01574 (16)	
H5BA	−0.0188	0.1701	0.3152	0.019*	
C6B	0.09823 (10)	−0.03191 (10)	0.35910 (9)	0.01469 (16)	
C7B	0.20549 (10)	−0.01329 (10)	0.39715 (9)	0.01585 (17)	
H7BA	0.2733	−0.0917	0.4283	0.019*	
C8B	0.32919 (10)	0.22659 (11)	0.43096 (9)	0.01633 (17)	
C9B	−0.30345 (11)	−0.00063 (12)	0.20815 (11)	0.0222 (2)	
H9BA	−0.3624	−0.0365	0.1894	0.033*	
H9BB	−0.2670	0.0658	0.1342	0.033*	
H9BC	−0.3583	0.0444	0.2743	0.033*	
O1W	0.34588 (8)	0.53871 (8)	0.45766 (7)	0.02077 (15)	
H2W1	0.3911	0.4524	0.4611	0.031*	
H1W1	0.3945	0.5902	0.3981	0.031*	
H2NA	0.8880 (18)	0.4499 (18)	0.0167 (16)	0.037 (4)*	
H2NB	0.3777 (17)	0.0311 (18)	0.4514 (15)	0.032 (4)*	
H3NA	0.6636 (16)	0.3855 (16)	0.3001 (14)	0.025 (4)*	
H3NB	0.7234 (15)	0.4214 (16)	0.3744 (14)	0.025 (4)*	
H3NC	0.2333 (17)	0.4172 (18)	0.4051 (15)	0.033 (4)*	
H3ND	0.1594 (16)	0.3384 (16)	0.3798 (14)	0.026 (4)*	
H1OA	0.4418 (18)	0.2630 (18)	−0.2614 (16)	0.036 (4)*	
H1OB	−0.0391 (18)	−0.3255 (18)	0.3063 (16)	0.037 (4)*	

### Atomic displacement parameters (Å^2^)

**Table:**

	*U*^11^	*U*^22^	*U*^33^	*U*^12^	*U*^13^	*U*^23^
S1A	0.01803 (11)	0.02175 (12)	0.01820 (11)	−0.01016 (9)	−0.00575 (8)	−0.00572 (9)
O1A	0.0214 (3)	0.0294 (4)	0.0160 (3)	−0.0126 (3)	−0.0040 (3)	−0.0087 (3)
O2A	0.0179 (3)	0.0289 (4)	0.0197 (3)	−0.0134 (3)	−0.0029 (3)	−0.0087 (3)
N1A	0.0144 (3)	0.0177 (4)	0.0181 (4)	−0.0063 (3)	−0.0058 (3)	−0.0050 (3)
N2A	0.0165 (3)	0.0214 (4)	0.0155 (3)	−0.0096 (3)	−0.0047 (3)	−0.0048 (3)
N3A	0.0176 (4)	0.0251 (4)	0.0174 (4)	−0.0100 (3)	−0.0029 (3)	−0.0079 (3)
C1A	0.0152 (4)	0.0199 (4)	0.0155 (4)	−0.0075 (3)	−0.0031 (3)	−0.0057 (3)
C2A	0.0166 (4)	0.0179 (4)	0.0149 (4)	−0.0063 (3)	−0.0049 (3)	−0.0057 (3)
C3A	0.0144 (4)	0.0174 (4)	0.0179 (4)	−0.0066 (3)	−0.0047 (3)	−0.0054 (3)
C4A	0.0162 (4)	0.0203 (4)	0.0156 (4)	−0.0079 (3)	−0.0031 (3)	−0.0048 (3)
C5A	0.0163 (4)	0.0197 (4)	0.0141 (4)	−0.0068 (3)	−0.0042 (3)	−0.0049 (3)
C6A	0.0142 (4)	0.0165 (4)	0.0160 (4)	−0.0052 (3)	−0.0051 (3)	−0.0047 (3)
C7A	0.0151 (4)	0.0193 (4)	0.0172 (4)	−0.0070 (3)	−0.0045 (3)	−0.0048 (3)
C8A	0.0147 (4)	0.0142 (4)	0.0167 (4)	−0.0042 (3)	−0.0058 (3)	−0.0045 (3)
C9A	0.0193 (4)	0.0321 (6)	0.0253 (5)	−0.0151 (4)	−0.0009 (4)	−0.0105 (4)
S1B	0.01952 (11)	0.01809 (12)	0.02886 (13)	−0.00806 (9)	−0.01069 (10)	−0.00520 (9)
O1B	0.0208 (3)	0.0155 (3)	0.0330 (4)	−0.0036 (3)	−0.0123 (3)	−0.0099 (3)
O2B	0.0178 (3)	0.0175 (3)	0.0280 (4)	−0.0032 (3)	−0.0118 (3)	−0.0089 (3)
N1B	0.0142 (3)	0.0186 (4)	0.0181 (4)	−0.0064 (3)	−0.0052 (3)	−0.0054 (3)
N2B	0.0157 (3)	0.0157 (4)	0.0236 (4)	−0.0053 (3)	−0.0086 (3)	−0.0049 (3)
N3B	0.0193 (4)	0.0159 (4)	0.0279 (4)	−0.0046 (3)	−0.0095 (3)	−0.0061 (3)
C1B	0.0148 (4)	0.0152 (4)	0.0201 (4)	−0.0028 (3)	−0.0063 (3)	−0.0065 (3)
C2B	0.0158 (4)	0.0141 (4)	0.0191 (4)	−0.0044 (3)	−0.0049 (3)	−0.0067 (3)
C3B	0.0146 (4)	0.0169 (4)	0.0164 (4)	−0.0057 (3)	−0.0047 (3)	−0.0059 (3)
C4B	0.0168 (4)	0.0152 (4)	0.0187 (4)	−0.0045 (3)	−0.0072 (3)	−0.0045 (3)
C5B	0.0170 (4)	0.0137 (4)	0.0183 (4)	−0.0050 (3)	−0.0058 (3)	−0.0044 (3)
C6B	0.0148 (4)	0.0156 (4)	0.0158 (4)	−0.0055 (3)	−0.0041 (3)	−0.0050 (3)
C7B	0.0150 (4)	0.0164 (4)	0.0182 (4)	−0.0050 (3)	−0.0054 (3)	−0.0051 (3)
C8B	0.0165 (4)	0.0172 (4)	0.0173 (4)	−0.0073 (3)	−0.0039 (3)	−0.0045 (3)
C9B	0.0193 (4)	0.0214 (5)	0.0322 (5)	−0.0022 (4)	−0.0141 (4)	−0.0104 (4)
O1W	0.0225 (4)	0.0180 (3)	0.0206 (3)	−0.0044 (3)	−0.0056 (3)	−0.0051 (3)

### Geometric parameters (Å, °)

**Table:**

S1A—C8A	1.6988 (10)	O1B—C2B	1.3668 (12)
O1A—C2A	1.3794 (11)	O1B—H1OB	0.820 (17)
O1A—H1OA	0.814 (17)	O2B—C3B	1.3633 (12)
O2A—C3A	1.3593 (12)	O2B—C9B	1.4324 (12)
O2A—C9A	1.4322 (13)	N1B—C7B	1.2836 (13)
N1A—C7A	1.2857 (12)	N1B—N2B	1.3824 (11)
N1A—N2A	1.3794 (12)	N2B—C8B	1.3373 (13)
N2A—C8A	1.3486 (12)	N2B—H2NB	0.841 (17)
N2A—H2NA	0.928 (17)	N3B—C8B	1.3292 (13)
N3A—C8A	1.3219 (13)	N3B—H3NC	0.832 (17)
N3A—H3NA	0.847 (16)	N3B—H3ND	0.875 (16)
N3A—H3NB	0.864 (15)	C1B—C2B	1.3816 (13)
C1A—C2A	1.3776 (13)	C1B—C6B	1.4016 (14)
C1A—C6A	1.4028 (13)	C1B—H1BA	0.9300
C1A—H1AA	0.9300	C2B—C3B	1.4037 (13)
C2A—C3A	1.3993 (14)	C3B—C4B	1.3905 (14)
C3A—C4A	1.3912 (13)	C4B—C5B	1.3888 (13)
C4A—C5A	1.3890 (14)	C4B—H4BA	0.9300
C4A—H4AA	0.9300	C5B—C6B	1.3966 (13)
C5A—C6A	1.3977 (14)	C5B—H5BA	0.9300
C5A—H5AA	0.9300	C6B—C7B	1.4547 (13)
C6A—C7A	1.4550 (13)	C7B—H7BA	0.9300
C7A—H7AA	0.9300	C9B—H9BA	0.9600
C9A—H9AA	0.9600	C9B—H9BB	0.9600
C9A—H9AB	0.9600	C9B—H9BC	0.9600
C9A—H9AC	0.9600	O1W—H2W1	0.8600
S1B—C8B	1.7090 (10)	O1W—H1W1	0.8531
			
C2A—O1A—H1OA	109.5 (12)	C3B—O2B—C9B	115.87 (8)
C3A—O2A—C9A	117.44 (8)	C7B—N1B—N2B	114.34 (8)
C7A—N1A—N2A	115.51 (8)	C8B—N2B—N1B	120.11 (8)
C8A—N2A—N1A	118.54 (8)	C8B—N2B—H2NB	119.9 (11)
C8A—N2A—H2NA	123.0 (10)	N1B—N2B—H2NB	119.9 (11)
N1A—N2A—H2NA	118.2 (10)	C8B—N3B—H3NC	118.1 (11)
C8A—N3A—H3NA	120.0 (10)	C8B—N3B—H3ND	118.5 (10)
C8A—N3A—H3NB	122.4 (10)	H3NC—N3B—H3ND	122.8 (15)
H3NA—N3A—H3NB	117.6 (14)	C2B—C1B—C6B	120.65 (9)
C2A—C1A—C6A	119.93 (9)	C2B—C1B—H1BA	119.7
C2A—C1A—H1AA	120.0	C6B—C1B—H1BA	119.7
C6A—C1A—H1AA	120.0	O1B—C2B—C1B	118.58 (9)
C1A—C2A—O1A	119.54 (9)	O1B—C2B—C3B	121.76 (9)
C1A—C2A—C3A	120.63 (9)	C1B—C2B—C3B	119.67 (9)
O1A—C2A—C3A	119.82 (8)	O2B—C3B—C4B	125.42 (9)
O2A—C3A—C4A	126.57 (9)	O2B—C3B—C2B	114.74 (9)
O2A—C3A—C2A	113.56 (8)	C4B—C3B—C2B	119.84 (9)
C4A—C3A—C2A	119.86 (9)	C5B—C4B—C3B	120.41 (9)
C5A—C4A—C3A	119.55 (9)	C5B—C4B—H4BA	119.8
C5A—C4A—H4AA	120.2	C3B—C4B—H4BA	119.8
C3A—C4A—H4AA	120.2	C4B—C5B—C6B	120.01 (9)
C4A—C5A—C6A	120.79 (9)	C4B—C5B—H5BA	120.0
C4A—C5A—H5AA	119.6	C6B—C5B—H5BA	120.0
C6A—C5A—H5AA	119.6	C5B—C6B—C1B	119.39 (9)
C5A—C6A—C1A	119.24 (9)	C5B—C6B—C7B	122.48 (9)
C5A—C6A—C7A	122.91 (8)	C1B—C6B—C7B	118.13 (9)
C1A—C6A—C7A	117.85 (9)	N1B—C7B—C6B	121.82 (9)
N1A—C7A—C6A	121.12 (9)	N1B—C7B—H7BA	119.1
N1A—C7A—H7AA	119.4	C6B—C7B—H7BA	119.1
C6A—C7A—H7AA	119.4	N3B—C8B—N2B	118.02 (9)
N3A—C8A—N2A	117.23 (9)	N3B—C8B—S1B	123.94 (8)
N3A—C8A—S1A	123.10 (7)	N2B—C8B—S1B	118.01 (7)
N2A—C8A—S1A	119.64 (7)	O2B—C9B—H9BA	109.5
O2A—C9A—H9AA	109.5	O2B—C9B—H9BB	109.5
O2A—C9A—H9AB	109.5	H9BA—C9B—H9BB	109.5
H9AA—C9A—H9AB	109.5	O2B—C9B—H9BC	109.5
O2A—C9A—H9AC	109.5	H9BA—C9B—H9BC	109.5
H9AA—C9A—H9AC	109.5	H9BB—C9B—H9BC	109.5
H9AB—C9A—H9AC	109.5	H2W1—O1W—H1W1	109.0
C2B—O1B—H1OB	109.3 (12)		
			
C7A—N1A—N2A—C8A	176.03 (9)	C7B—N1B—N2B—C8B	−175.36 (9)
C6A—C1A—C2A—O1A	−179.88 (9)	C6B—C1B—C2B—O1B	177.67 (9)
C6A—C1A—C2A—C3A	0.19 (15)	C6B—C1B—C2B—C3B	−1.69 (15)
C9A—O2A—C3A—C4A	−3.53 (16)	C9B—O2B—C3B—C4B	−1.14 (14)
C9A—O2A—C3A—C2A	177.37 (9)	C9B—O2B—C3B—C2B	178.05 (9)
C1A—C2A—C3A—O2A	179.06 (9)	O1B—C2B—C3B—O2B	1.74 (14)
O1A—C2A—C3A—O2A	−0.87 (14)	C1B—C2B—C3B—O2B	−178.92 (9)
C1A—C2A—C3A—C4A	−0.10 (15)	O1B—C2B—C3B—C4B	−179.02 (9)
O1A—C2A—C3A—C4A	179.97 (9)	C1B—C2B—C3B—C4B	0.33 (15)
O2A—C3A—C4A—C5A	−178.74 (10)	O2B—C3B—C4B—C5B	179.85 (9)
C2A—C3A—C4A—C5A	0.30 (15)	C2B—C3B—C4B—C5B	0.69 (15)
C3A—C4A—C5A—C6A	−0.61 (15)	C3B—C4B—C5B—C6B	−0.35 (15)
C4A—C5A—C6A—C1A	0.70 (15)	C4B—C5B—C6B—C1B	−1.00 (15)
C4A—C5A—C6A—C7A	−179.96 (10)	C4B—C5B—C6B—C7B	178.61 (9)
C2A—C1A—C6A—C5A	−0.49 (15)	C2B—C1B—C6B—C5B	2.03 (15)
C2A—C1A—C6A—C7A	−179.86 (9)	C2B—C1B—C6B—C7B	−177.59 (9)
N2A—N1A—C7A—C6A	179.81 (9)	N2B—N1B—C7B—C6B	−178.82 (9)
C5A—C6A—C7A—N1A	3.67 (16)	C5B—C6B—C7B—N1B	−3.38 (15)
C1A—C6A—C7A—N1A	−176.98 (9)	C1B—C6B—C7B—N1B	176.23 (9)
N1A—N2A—C8A—N3A	0.26 (14)	N1B—N2B—C8B—N3B	0.04 (14)
N1A—N2A—C8A—S1A	178.63 (7)	N1B—N2B—C8B—S1B	−178.29 (7)

### Hydrogen-bond geometry (Å, °)

**Table:**

*D*—H···*A*	*D*—H	H···*A*	*D*···*A*	*D*—H···*A*
O1W—H2W1···S1B	0.86	2.28	3.1257 (9)	169
O1W—H1W1···O1A^i^	0.85	1.95	2.7955 (12)	173
N2A—H2NA···S1A^ii^	0.929 (18)	2.450 (18)	3.3732 (9)	172.6 (18)
N2B—H2NB···S1B^iii^	0.842 (19)	2.571 (18)	3.4055 (10)	171.2 (18)
N3A—H3NA···N1A	0.847 (19)	2.258 (15)	2.6129 (14)	105.4 (12)
N3A—H3NB···O1W^iv^	0.864 (16)	2.000 (15)	2.8408 (12)	164.0 (17)
N3B—H3NC···O1W	0.833 (19)	2.399 (19)	3.1492 (14)	150.2 (17)
N3B—H3ND···N1B	0.875 (19)	2.288 (17)	2.6554 (14)	105.2 (13)
O1A—H1OA···O2A	0.81 (2)	2.185 (18)	2.6292 (12)	114.5 (15)
O1B—H1OB···S1A^v^	0.82 (2)	2.685 (19)	3.2346 (10)	125.9 (16)
O1B—H1OB···O2B	0.82 (2)	2.251 (19)	2.6949 (13)	114.4 (16)
C1B—H1BA···O1W^vi^	0.93	2.40	3.3140 (14)	169
C9B—H9BA···O2A^vii^	0.96	2.51	3.2286 (15)	131

Symmetry codes: (i) −*x*+1, −*y*+1, −*z*; (ii) −*x*+2, −*y*+1, −*z*; (iii) −*x*+1, −*y*, −*z*+1; (iv) −*x*+1, −*y*+1, −*z*+1; (v) *x*−1, *y*−1, *z*; (vi) *x*, *y*−1, *z*; (vii) −*x*, −*y*, −*z*.
